# Does pyrethroid exposure lower human semen quality? a systematic review and meta-analysis

**DOI:** 10.3389/ftox.2024.1395010

**Published:** 2024-05-30

**Authors:** Roland Eghoghosoa Akhigbe, Precious Adeoye Oyedokun, Tunmise Maryanne Akhigbe, Suliat Adenike, Ayoola Abimbola Oladipo, Jennifer Rose Hughes

**Affiliations:** ^1^ Department of Physiology, Ladoke Akintola University of Technology, Ogbomoso, Nigeria; ^2^ Reproductive Biology and Toxicology Research Laboratory, Oasis of Grace Hospital, Osogbo, Nigeria; ^3^ Breeding and Genetics Unit, Department of Agronomy, Osun State University, Osogbo, Nigeria; ^4^ Acrolytics LLC, Fort Collins, CO, United States

**Keywords:** endocrine disruptor, environmental toxicant, male infertility, oxidative stress, pyrethroids, semen

## Abstract

**Background:** Pyrethroids are natural organic compounds extracted from flowers of pyrethrums and commonly used as domestic and commercial insecticides. Although it is effective in insect and parasitic control, its associated toxicity, including spermotoxicity, remains a challenge globally. Currently, the available reports on the effect of pyrethroids on semen quality are conflicting, hence an evaluation of its detrimental effect is pertinent. This study conducts a detailed systematic review and meta-analysis of the effects of pyrethroids on sperm quality.

**Materials and methods:** The present study was performed according to Preferred Reporting Items for Systematic Reviews and Meta-Analyses (PRISMA) guidelines. Using a pre-defined strategic protocol, an internet search was done using combined text words. The criteria for eligibility were selected based on Population, Exposure, Comparator, Outcome, and Study Designs (PECO) framework, and relevant data were collected. Appraisal was done using The Office of Health Assessment and Translation (OHAT) tool for the evaluation of the Risk of Bias and the Grading of Recommendations Assessment, Development, and Evaluation (GRADE) Working Group guidelines for the certainty of evidence. A quantitative meta-analysis was conducted with the Review Manager (RevMan).

**Results:** Only 12 out of the 4, 050 studies screened were eligible for inclusion in this study. The eligible studies were from China (4), Japan (3), Poland (3), and United States (2). All the eligible studies were cross-sectional. A total of 2, 050 male subjects were included in the meta-analysis. Pyrethroid exposure significantly reduced sperm motility. Region-stratified subgroup analyses revealed that pyrethroid significantly reduced sperm motility among men in Poland and United States, and decreased sperm count among men in Japan. Pyrethroid exposure also reduced sperm concentration among men in Poland but increased sperm concentration among men in the United States.

**Conclusion:** Although the study revealed inconsistent evidence on the detrimental effect of pyrethroids on semen quality, the findings showed that pyrethroids have deleterious potentials on sperm motility, count, and concentration. Studies focusing on the assessment of semen quality in pyrethroid-exposed men, especially at specific varying levels of exposure, and employing prospective cohort studies or controlled cross-sectional designs are recommended.

## Introduction

Pyrethroids are natural organic compounds extracted from flowers of pyrethrums and commonly used as domestic and commercial insecticides (Hodoșan et al., 2023). Though innocuous to humans in household concentration (e.g., 0.05 mg/kg/day of cypermethrin (Woolen et al., 1992), pyrethroids are toxic to bees, fishes, gadflies, dragonflies, and mayflies with mounting evidence of effectiveness in controlling malaria outbreaks through mosquitoes (Ramchandra et al., 2019; Galadima et al., 2021). The most common types of pyrethroids (cyfluthrin, cyphenothrin, cypernothrin, fenvalerate, and permethrin) are active chemicals in insect-control products such as Baygon, Tempo SC, K2000, Temprid, and Fumakilla Vape Aerosol. Sources of pyrethroid poisoning through skin contact, ingestion, or inhalation may include crop protection practices, veterinary medicine for parasitic infestations, or contact with soaked mosquito nets, sprays, or gels (Hołyńska-Iwan and Szewczyk-Golec, 2020). Permethrin-impregnated nets have been recommended by the World Health Organization as a control measure to control Zika virus infection (WHO, 2016).

Pyrethroid poisoning has been shown to cause facial paresthesia, muscle twitching, respiratory irritation (Saillenfait et al., 2015), seizures (Hansen et al., 2017), bleeding (Saillenfait et al., 2015), coma (Bradberry et al., 2005), pulmonary edema (Vorselaars et al., 2021), and impairment of early social-emotional and language development (Pitzer et al., 2021). Sustained exposure to pyrethroids has been implicated in the development of neurodegenerative diseases including Alzheimer’s dementia, Parkinsonism, and amyotrophic lateral sclerosis (Hansen et al., 2017). In an experimental study in rats, pyrethroid (30 mg/m^3^ of a combination of 0.02%/w/w imiprothrin, 0.03%/w/w d-phenothrin. And 0.10%/w/w d-trans allethrin, Mortein^®^) was observed to reduce the mean arterial pressure but increase the pulse pressure (Saka et al., 2012) with no significant alterations of hematological and hemostatic variables (Saka et al., 2011).

When analyzing reproductive outcomes, pyrethroid exposure creates inconsistent outcomes. According to Saillenfait et al. (2015), exposure to pyrethroids was associated with impaired reproductive functions, i.e., reduced semen quality, and sperm DNA integrity, and altered reproductive hormones; while Lifeng et al. (2006a) observed no significant alteration in semen quality in men exposed to pyrethroid. An earlier report by Xia et al. (2004b) also demonstrated no significant alteration in the conventional semen variables following exposure to fenvalerate, a common pyrethroid pesticide. In their systematic review (SR) and meta-analysis (MA) of rodent studies, Zhang et al. (2021) reported that pyrethroid exposure led to a decrease in sperm count, sperm morphology, sperm motility, and epididymal weight, specifically in rats and mice. Similarly, in the meta-analysis by Zhang et al. (2021), exposure to pyrethroid at gestation and lactation caused significant suppression of reproductive capacity in male F1 offspring. The adult F1 males had reduced epididymal weight, lower sperm count, and lower sperm motility. The sperm from these males had higher levels of lipid peroxidation, and suppression of endogenous antioxidants: glutathione, superoxide dismutase, and catalase (Zhang et al., 2021).

The evidence that pyrethroid exposure in rodents reduces male fertility measures is consistent between animal models and exposure levels. However, the available reports on the effect of pyrethroid exposure on human semen quality are scanty and there is no SR and MA on the impact of pyrethroid exposure on human semen quality. Since it is important to translate findings in experimental rodents to humans with utmost care and the reports of Zhang et al. (2021) were in rodents, this study was designed to evaluate the effect of pyrethroid exposure on human semen quality through a (SR) and (MA). The research question: **“**Does pyrethroid exposure lower human semen quality?” was formed in line with the PECOS (Population, Exposure, Comparators, Outcome, and Study Design) statement.

## Materials and methods

The Preferred Reporting Items for Systematic Reviews and Meta-Analysis (PRISMA) guide (Hamed et al., 2023) was adopted to conduct this review.

### Literature search

Online search was performed in EMBASE, Pubmed/MEDLINE, and Web of Science databases through 31 January 2024, by using search items related to “pyrethroid” and “semen” or “sperm”. In addition, citation-chasing techniques were employed to identify relevant papers. The reference lists and backward and forward citations of the collected papers were screened (Booth, 2008; European Network for Health Technology Assessment EUnetHTA, 2019). Two authors (PAO and TMA) independently screened the titles and abstracts of the publications and evaluated the full text for eligibility. In a case of dispute, both authors reviewed the full text through a consensus-based discussion. When disagreement persisted, a third author (REA) resolved the dispute.

### Eligibility assessment and study selection

The eligibility criteria for studies to be included were determined by the Population, Exposure, Comparator, Outcome, and Study Designs (PECO) framework (Hamed et al., 2023).

The inclusion criteria include studies among male adults who were between 18 and 50 years old and have been exposed to environmental or occupational pyrethroid alone or in combination with a specified environmental toxicant. Also, the studies should be case-control, cross-sectional, cohort, or ecological, that appropriately answer the question “What is the effect of pyrethroid exposure on human semen quality?”, and report relevant parameters demonstrating the impact of pyrethroid on semen quality, using the mean and standard deviation or any other form from which the mean and standard deviation can be calculated. Studies without controls were compared with the standard WHO reference values.

*In vitro* studies and studies on animal models, studies on prenatal pyrethroid exposure, or exposure to environmental toxicants apart from pyrethroid were excluded. In addition, studies that did not document the actual values of the variables of interest in the form of mean and standard deviation or any other form from which the mean and standard deviation can be calculated were screened out. Furthermore, studies documenting self-reported reproductive health outcomes, case studies, review articles, commentaries, letters and editorials, conference abstracts, preprint, degree thesis, and retracted papers were excluded. However, language and publication date restrictions were not applied.

### Data extraction

The data that were collected from the eligible studies were the last name (surname) of the first author and year of publication, study design, country where the study was conducted, sample size, age of the participants, type of pyrethroid participants were exposed to, duration of exposure, sexual/ejaculation abstinence period, mean and standard deviation of variables of interest. Where the mean and standard deviation were not provided, values from which the mean and standard deviation can be calculated were extracted.

### Assessment of the risk of bias

The quality of the studies included was assessed using The Office of Health Assessment and Translation (OHAT) Risk of Bias (RoB) tool (OHAT, 2015). Each of the six domains assessed was adjudged low risk of bias and scored one or high risk of bias which did not attract any score.

### Assessment of the certainty of the evidence

The overall certainty of the evidence was assessed using the Grading of Recommendations Assessment, Development, and Evaluation (GRADE) Working Group guidelines (GRADE, 2014) to adjudge each study as one with a high, moderate, low, or very low level of confidence (Rooney et al., 2014).

### Synthesis of evidence, meta-analysis, and sensitivity analysis

Both qualitative and quantitative methods were used to generate the evidence from all of the research. In the qualitative approach, the inclusion criteria for eligibility were applied. Review Manager (RevMan) software (version 5.4.1) was used to perform quantitative meta-analysis. To ascertain the heterogeneity of the studies or the percentage of total variance across studies, the mean difference (MD) of the reported variables was pooled at 95% confidence intervals (95% CIs) and the *p*-value and I-square statistic (I2) in the pooled analyses were utilized. When *the p*-value was <0.1 or the I^2^ -value >50% which suggests a significant heterogeneity, a random-effects model was used, but when the *p*-value was ≥0.1 or the I^2^ -value ≤50%, indicating low heterogeneity, a fixed-effects model was used. The heterogeneity indicates differences within individual samples, between samples, and between experimental results. Sensitivity analyses were conducted by removing the study with the most weight, studies at high risk, and studies with low or very low confidence in the evidence. Subgroup analyses were conducted by grouping studies from the same region together.

Furthermore, publication bias for studies included per variable of interest was visually assessed using the funnel plot created by Review Manager (RevMan) software (Hamed et al., 2023). The asymmetry of the funnel plot suggests possible publication bias. Values are presented as the mean difference and 95% of the confidence interval (CI). The mean difference is obtained as the difference between the values of the pyrethroid-exposed and the control.

## Results

### Study selection and characteristics of eligible studies

**FIGURE 1:**
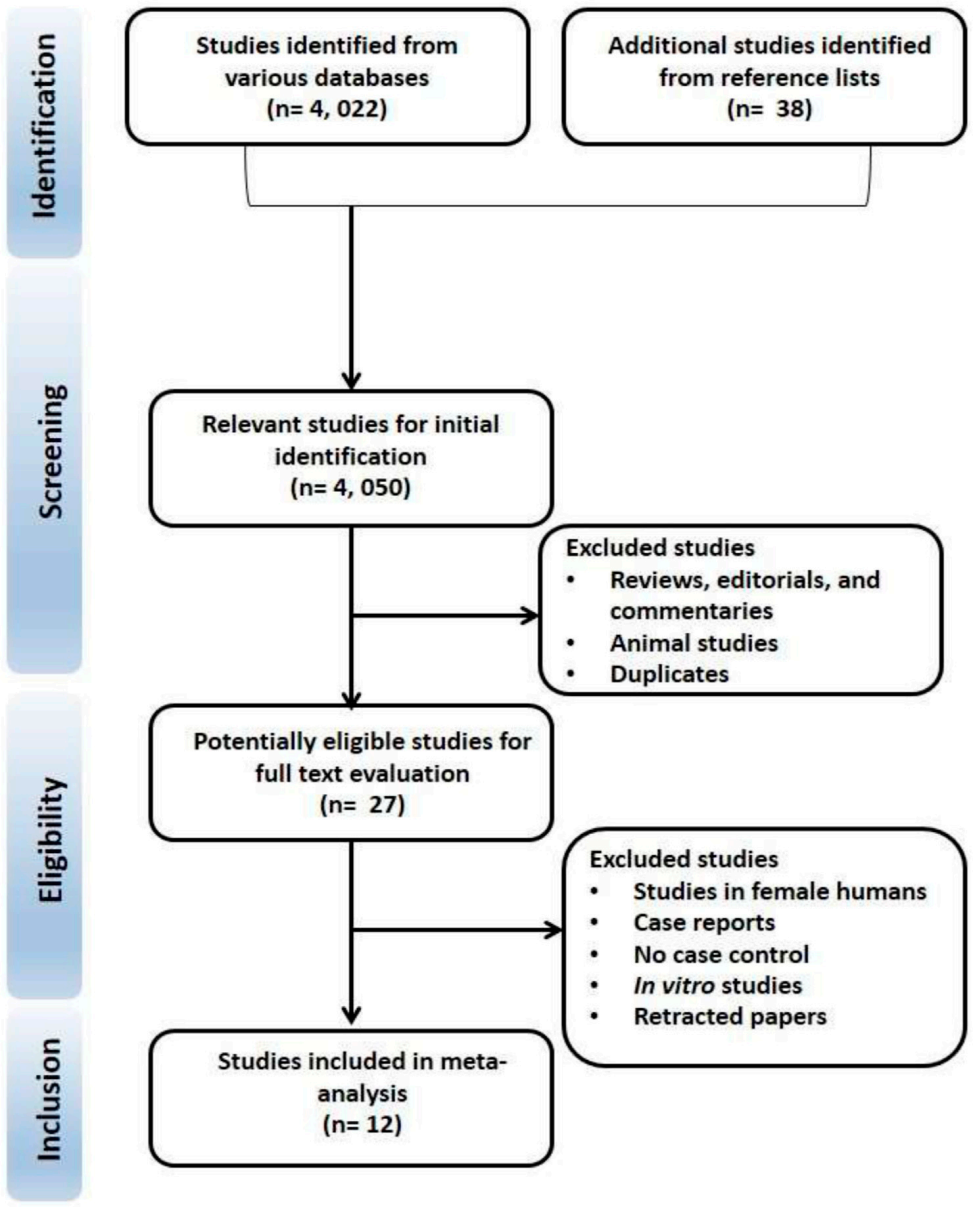
PRISMA flow chart for the selection of eligible studies.

The PRISMA flow chart is presented in Figure 1. We retrieved 4, 022 studies from the search and an additional 38 from the citation-chasing technique, making a total of 4, 050 studies. After screening the review studies, editorials, commentaries, animal studies, and duplicates, 27 studies were left. Fifteen more papers were excluded on further screening, leaving 12 papers that fully met the eligibility criteria.

The summary of the characteristics of the 12 eligible studies is provided in Table 1. Overall, the included studies were published between 2004 and 2020, with most from China (4), Japan (3), and Poland (3). The remaining two were from the United States. All the studies were cross-sectional and one (Kamijima et al., 2004) reported findings in two seasons, in the summer and winter. There were a total of 2, 050 male subjects between age 18 and 50 years. Three of the studies (Xia et al., 2004a; Bian et al., 2004; Linfeng et aL, 2006b) were on fenvalerate exposure, eight (Meeker JD. et al., 2008; Toshima et al., 2012; Young HA. et al., 2013; Radwan et al., 2014b; Imai et al., 2014; Jurewicz et al., 2015; Radwan et al., 2015; Hu et al., 2020) were on unspecified environmental pyrethroids, while one (Kamijima et al., 2004) was on combined organophosphate and pyrethroid.

**TABLE 1:** Characteristics of the eligible studies included in the meta-analysis.

References	Study design	Country	Examined population	Age (years)	Exposure	Duration of exposure	Outcomes/variables measured		
							Semen	Abstinence period (days)	Others
Bian et al. (2004)	Cross-sectional	China	Exposed: 21, Internal control: 23, External control: 19	22–45 years	Fenvalerate	12 months or at least 6 continuous months	Sperm concentration and motility	3–5 days	Comet assay, Tunel assay
Hu et al. (2020)	Cross-sectional	China	346	20–50 years	Environmental pyrethroids	Not specified	Semen volume, sperm concentration, sperm count, sperm morphology, sperm motility	≤3 days (28.6% of the participants), 3–5 days (33.8 of the participants), >5 days (37.6 of the participants)	
Imai et al. (2014)	Cross sectional	Japan	322	18–24 years	Environmental pyrethroids	Not specified	Semen volume, sperm concentration, sperm count, sperm motility	78.1 ± 32.3 h (M±SD)	-
Jurewicz et al. (2015)	Cross Sectional	Poland	286	32.22, 4.45 (mean, SD)	Environmental pyrethroid	Not specified	Sperm concentration, motility, abnormal morphology	<3 (4.90% of the participants), 3–7 (74.13% of the participants), >7 (5.94% of the participants), and missing data (15.03% of the participants)	DNA fragmentation index
Kamijima et al., 2004	Cross Sectional	Japan	Exposed: 15 in summer and 14 in winter Control: 16 in summer and 15 in winter	Exposed: 33.8, 7.0 (mean, SD)	OP and pyrethroid insecticide	5.6, 5.8	Semen volume, sperm concentration, sperm count, sperm motility, sperm viability	4.5, 2 days (summer), 4.2, 1.4 days (winter) (median, range)	
				Unexposed: 34, 7.5 (mean, SD)					
Linfeng et al. (2006a)	Cross sectional	China	Exposed: 32Unexposed: 46	21–42 years	Fenvalerate	Not specified	Semen volume, pH, Motility, Concentration, Sperm count, liquefying time, viscidity	3 days	
Meeker et al. (2008b)	Cross-sectional	United States of America	207	35.7, 5.3 (mean, SD)	Environmental pyrethroid	Not specified	Sperm concentration, motility, and normal morphology	≤2(27% of the participants), 3 (29% of the participants), 4 (19% of the participants), 5 (11% of the participants), ≥6 (14% of the participants)	CASA parameters, DNA damage using comet
Radwan et al. (2014a)	Cross-sectional	Poland	334	32.24, 4.43 (mean, SD)	Environmental pyrethroid	Not specified	Sperm concentration, motility, and abnormal morphology	<3 (4.79% of the participants), 3–7 (73.95% of the participants), >7 (5.99% of the participants), and missing data (15.27% of the participants)	CASA parameters
Radwan et al. (2015)	Cross-sectional	Poland	195	32.2, 4.7, (mean, SD)	Environmental pyrethroid	Not specified	Sperm concentration, motility, and abnormal morphology	<3 (12.31% of the participants), 3–7 (71.28% of the participants), >7 (16.41% of the participants)	Aneuploidy
Toshima et al. (2012)	Cross-sectional	Japan	42	36.8, 5.4, (mean, SD)	Environmental pyrethroid	Not specified	Semen volume, concentration, motility, and	4.8, 2.0 (mean, SD)	
Xia et al. (2004a)	Cross Sectional	China	Exposed: 12	21–42 years	Fevalerate	Not specified	Semen volume, Sperm motility, Concentration, Sperm count, sperm abnormality	3 days	
			Unexposed: 30						
Young et al. (2013a)	Cross-sectional	United States of America	75	35.3, 5.1 (mean, SD)	Environmental pyrethroid	Not specified	Sperm concentration, motility, and normal morphology	≤2(25.3% of the participants), 3–4 (48% of the participants), ≥5 (26.7% of the participants)	Aneuploidy

### Assessment of the RoB and certainty of evidence

The results of the quality of the studies assessed by the OHAT RoB tool are presented in Table 2. Three of the studies (Kamiji ma et al., 2004; Xia et al., 2004b; Linfeng et aL, 2006a) had 7/9 indicating low RoB, while one (Bian et al., 2004) had 6/9 and eight (Meeker JD. et al., 2008; Toshima et al., 2012; Young H. A. et al., 2013; Radwan et al., 2014a; Imai et al., 2014; Jurewicz et al., 2015; Radwan et al., 2015; Hu et al., 2020) had 4/9 indicating moderate RoB. Overall, no study was adjudged high RoB.

Using the GRADE guideline for the certainty in the body of evidence, ten of the studies (Bian et al., 2004; Kamijima et al., 2004; Xia et al., 2004b; Linfeng et aL, 2006b; Meeker J. D. et al., 2008; Radwan et al., 2014b; Imai et al., 2014; Jurewicz et al., 2015; Radwan et al., 2015; Hu et al., 2020) were deemed to have moderate certainty of the evidence, while two (Toshima et al., 2012; Young HA. et al., 2013) had low certainty of evidence (Table 3).

### Quantitative synthesis and sensitivity analysis

#### Ejaculate volume

**FIGURE 2:**
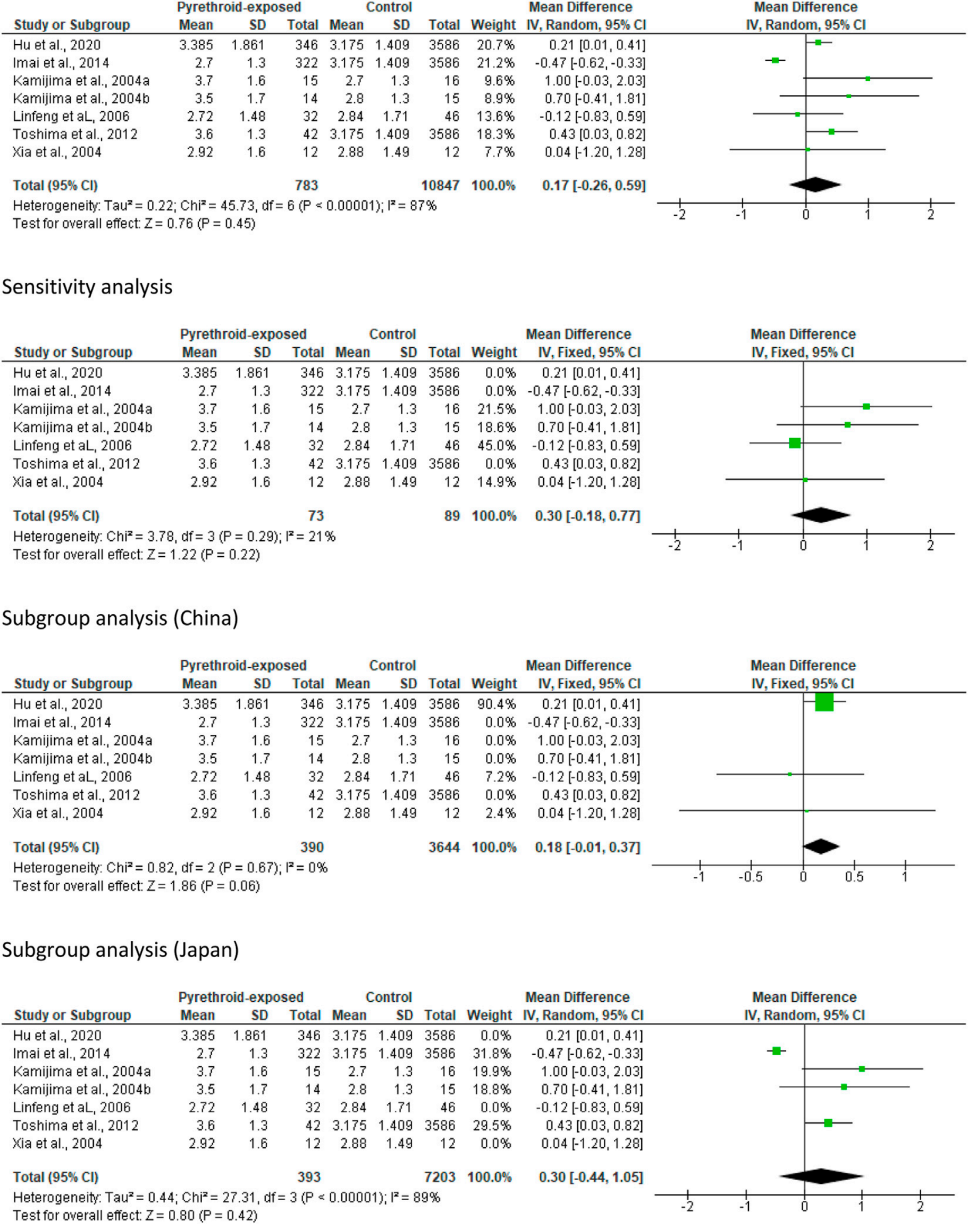
Pyrethroid exposure does not alter ejaculate volume (mL).

Analysis of all eligible studies revealed that pyrethroid did not alter ejaculate volume (MD 0.17 [95% CI: 0.26, 0.59] *p* = 0.45). There was a significant inter-study heterogeneity (I^2^ = 87%; *X* ^2^ *p <* 0.00001). After a sensitivity analysis, it was also observed that pyrethroid did not significantly alter ejaculate volume (MD 0.30 [95% CI: 0.18, 0.77] *p* = 0.22), and the inter-study heterogeneity was not significant (I^2^ = 21%; *X* ^2^ *p <* 0.29). Conversely, analyses stratified by region showed that pyrethroid exposure did not significantly alter ejaculate volume in Chinese subjects (MD 0.18 [95% CI: 0.01, 0.37] *p* = 0.06) and there was no significant inter-study heterogeneity (I^2^ = 0%; *X* ^2^ *p <* 0.67). More so, the subgroup analysis of the study from Japan revealed that pyrethroid did not alter ejaculate volume (MD 0.30 [95% CI: 0.44, 1.05] *p* = 0.42) with a significant inter-study heterogeneity (I^2^ = 89%; *X* ^2^ *p <* 0.00001) (Figure 2).

**TABLE 2:** Risk of bias assessment of the eligible studies.

Study	Selection of exposed cohort	Selection of non-exposed cohort	Assessment of exposure	Demonstration of outcome	Comparability (basics)	Comparability (others)	Assessment outcome	Length of follow-up	Adequacy of follow-up	Total
Bian et al. (2004)	1	1	-	1	1	1	1	-	-	6/9
Hu et al. (2020)	1	-	1	1	-	-	1	-	-	4/9
Imai et al. (2014)	1	-	1	1	-	-	1	-	-	4/9
Jurewicz et al. (2015)	1	-	1	1	-	-	1	-	-	4/9
Kamijima et al., 2004	1	1	1	1	1	1	1	-	-	7/9
Linfeng et al. (2006a)	1	1	1	1	1	1	1	-	-	7/9
Meeker et al. (2008a)	1	-	1	1	-	-	1	-	-	4/9
Radwan et al. (2014b)	1	-	1	1	-	-	1	-	-	4/9
Radwan et al. (2015)	1	-	1	1	-	-	1	-	-	4/9
Toshima et al. (2012)	1	-	1	1	-	-	1	-	-	4/9
Xia et al. (2004b)	1	1	1	1	1	1	1	-	-	7/9
Young et al. (2013b)	1	-	1	1	-	-	1	-	-	4/9

**TABLE 3:** Confidence of the body of evidence in the eligible studies.

Study	Initial confidences	Decreasing	Increasing	Final confidence
Bian et al. (2004)	Moderate	-	-	Moderate
Hu et al. (2020)	Low	-	+	Moderate
Imai et al. (2014)	Low	-	+	Moderate
Jurewicz et al. (2015)	Low	-	+	Moderate
Kamijima et al., 2004	Moderate	-	-	Moderate
Linfeng et al. (2006b)	Moderate	-	-	Moderate
Meeker et al. (2008b)	Low	-	+	Moderate
Radwan et al. (2014a)	Low	-	+	Moderate
Radwan et al. (2015)	Low	-	+	Moderate
Toshima et al. (2012)	Low	-	_	Low
Xia et al. (2004a)	Moderate	-	-	Moderate
Young et al. (2013a)	Low	-	_	Low

no effect, +: increased.

#### Sperm motility

**FIGURE 3:**
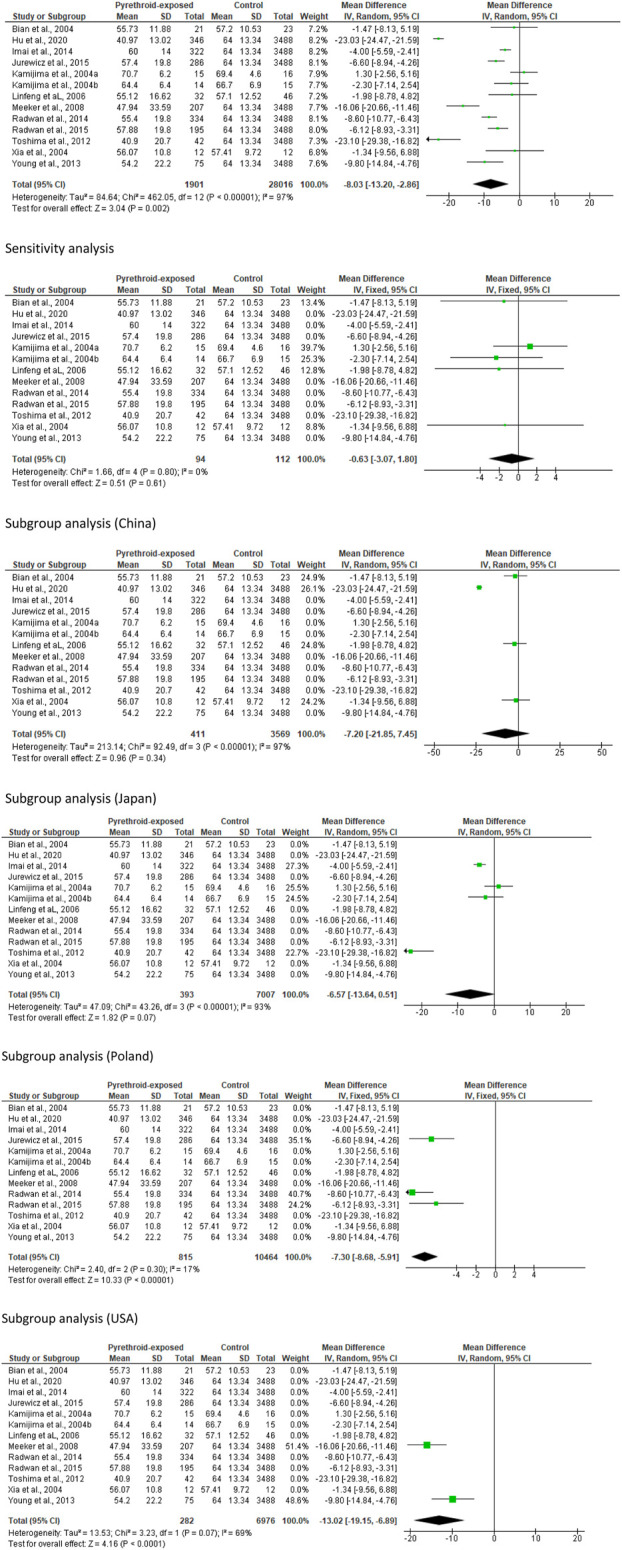
Pyrethroid exposure has mixed effects on sperm motility (%).

Thirteen studies from twelve published papers were included in the evaluation of the effect of pyrethroid on sperm motility. It was observed that pyrethroid significantly reduced sperm motility (MD -8.03 [95% CI: 13.20, −2.86] *p =* 0.002). Also, there was a significant inter-study heterogeneity (I^2^ = 97%; *X* ^2^ *p <* 0.00001). After a sensitivity analysis, it was also observed that pyrethroid did not significantly alter sperm motility (MD -0.63 [95% CI: 3.07, 1.80] *p* = 0.61), and the inter-study heterogeneity was not significant (I^2^ = 0%; *X* ^2^ *p =* 0.80). After the region-stratified analyses, it was observed that pyrethroid did not significantly lower sperm motility among men in China (MD -7.20 [95% CI: 21.85, 7.45] *p* = 0.34) and there was a significant inter-study heterogeneity (I^2^ = 97%; *X* ^2^ *p <* 0.00001). Also, pyrethroid did not significantly reduce sperm motility among men in Japan (MD -6.57 [95% CI: 13.64, 0.51] *p* < 0.00001), and the inter-study heterogeneity was significant (I^2^ = 93%; *X* ^2^ *p <* 0.00001). However, pyrethroid significantly reduced sperm motility among men in Poland (MD -7.30 [95% CI: 8.68, −5.91] *p* < 0.00001) and USA (MD -13.02 [95% CI: 19.15, −6.89] *p* < 0.0001). There was no significant inter-study heterogeneity in the studies conducted in Poland (I^2^ = 17%; *X* ^2^ *p =* 0.30) but this existed in the United States (I^2^ = 69%; *X* ^2^ *p =* 0.07) (Figure 3).

#### Sperm viability

**FIGURE 4:**
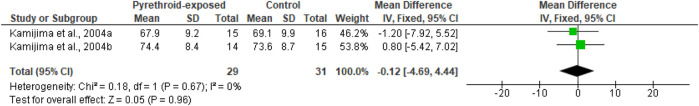
Pyrethroid exposure does not alter sperm viability.

Evaluation of the effect of pyrethroid on sperm viability revealed that pyrethroid did not significantly alter sperm viability (MD -0.12 [95% CI: 4.69, 4.44] *p =* 0.96). In addition, the inter-study heterogeneity was not significant (I^2^ = 0%; *X* ^2^ *p =* 0.67) (Figure 4). No sensitivity nor subgroup analysis was performed because the available human studies on the effect of pyrethroid exposure on sperm viability were scanty.

#### Sperm count

**FIGURE 5:**
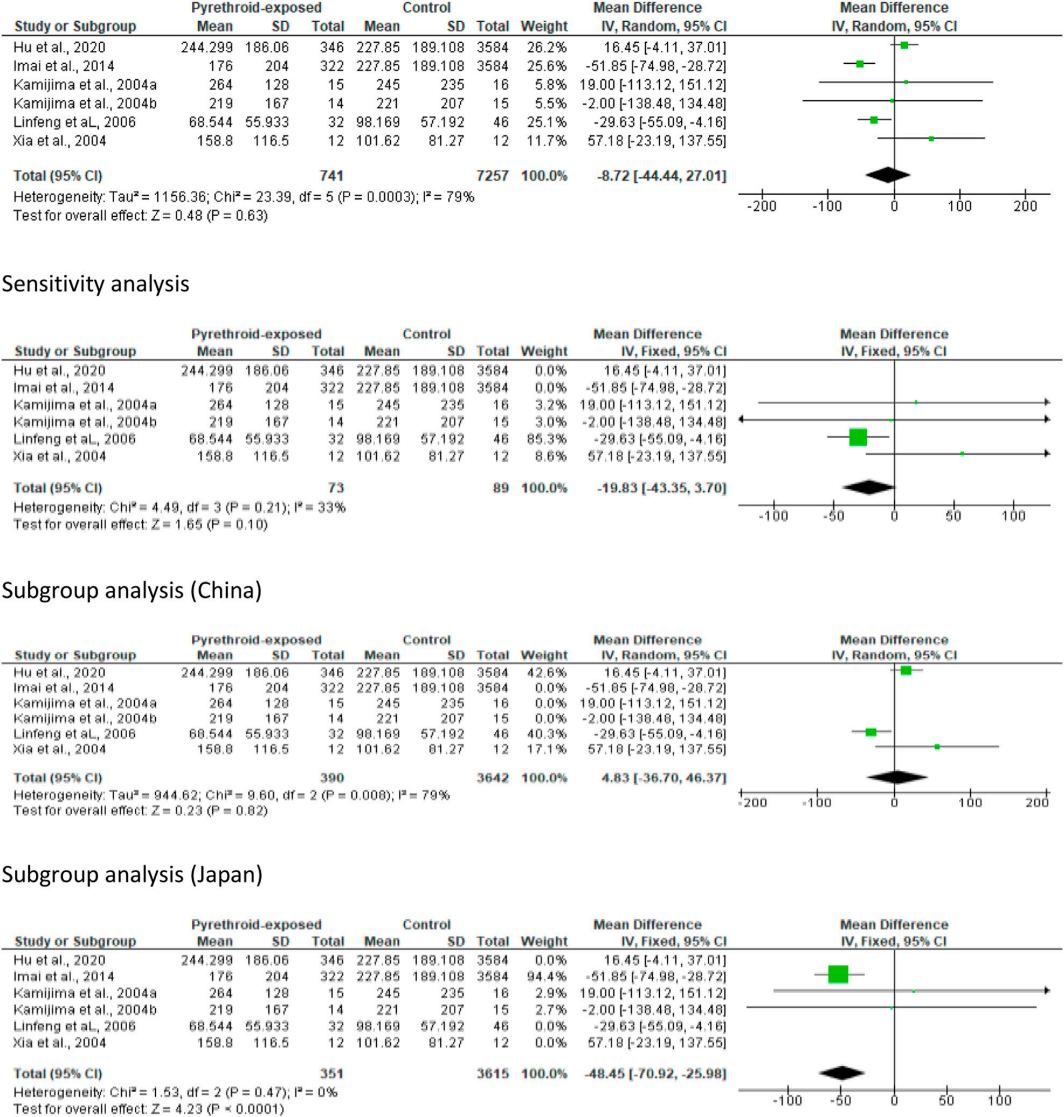
Sperm count is lowered in a region-specific manner after pyrethroid exposure.

With regards to the total number of sperm, we found out that pyrethroid did not significantly reduce sperm count (MD -8.72 [95% CI: 44.44, 27.01] *p =* 0.63). However, there was a significant inter-study heterogeneity (I^2^ = 79%; *X* ^2^ *p =* 0.0003). In sensitivity analysis, pyrethroid did not significantly alter sperm count (MD -19.83 [95% CI: 43.35, 3.70] *p* = 0.10), and the inter-study heterogeneity was not significant (I^2^ = 33%; *X* ^2^ *p =* 0.21). A similar pattern was observed after the region-stratified analysis among men in China, where it was observed that pyrethroid did not alter sperm count (MD -4.83 [95% CI: 36.70, 46.37] *p* = 0.82), but there was a significant inter-study heterogeneity (I^2^ = 79%; *X* ^2^ *p =* 0.008). Nonetheless, among men in Japan, pyrethroid significantly reduced sperm count (MD -48.45 [95% CI: 70.92, −25.98] *p* < 0.00001) and the inter-study heterogeneity was not significant (I^2^ = 0%; *X* ^2^ *p =* 0.47) (Figure 5).

#### Sperm concentration

**FIGURE 6:**
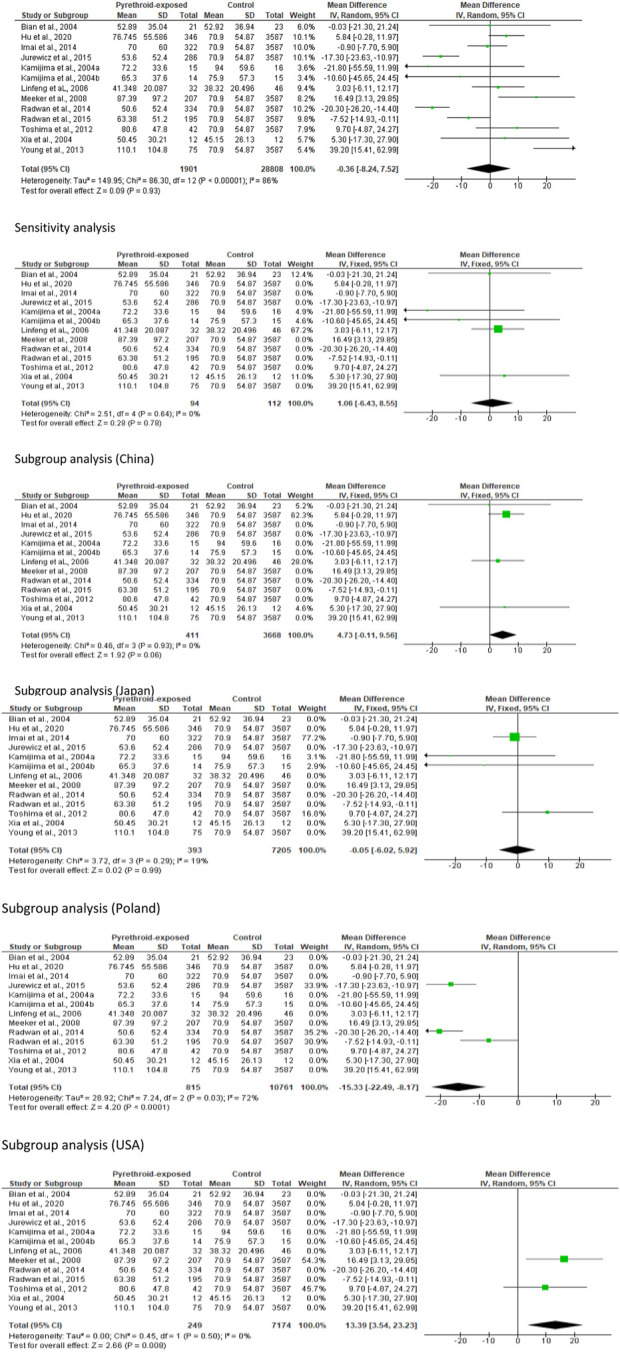
Pyrethroid exposure alters sperm concentration in a region-specific manner.

In the meta-analysis evaluating the effect of pyrethroid on sperm concentration, we found no significant change (MD -0.36 [95% CI: 8.24, 7.52] *p =* 0.93). Moreover, there was a significant inter-study heterogeneity (I^2^ = 86%; *X* ^2^ *p <* 0.00001). In sensitivity analysis, pyrethroid did not significantly reduce sperm concentration (MD 1.06 [95% CI: 6.43, 8.55] *p* = 0.78), and the inter-study heterogeneity was also not significant (I^2^ = 0%; *X* ^2^ *p =* 0.64). When the data were subjected to subgroup analysis, we observed that pyrethroid did not also significantly affect sperm concentration among men in China (MD 4.73 [95% CI: 0.11, 9.56] *p* = 0.06), and there was no significant inter-study heterogeneity (I^2^ = 0%; *X* ^2^ *p =* 0.93). A similar effect was observed among men in Japan (MD -0.05 [95% CI: 6.02, 5.92] *p* = 0.99) and the inter-study heterogeneity was not significant (I^2^ = 19%; *X* ^2^ *p =* 0.29). A significant decrease in sperm concentration was observed among men in Poland (MD -15.33 [95% CI: 22.49, −8.17] *p* < 0.0001) but the inter-study heterogeneity was significant (I^2^ = 72%; *X* ^2^ *p =* 0.03), while there was a significant increase in sperm concentration among men in United States following pyrethroid exposure (MD 13.39 [95% CI: 3.54, 23.23] *p* = 0.008) and the inter-study heterogeneity was not significant (I^2^ = 0%; *X* ^2^ *p =* 0.50) (Figure 6).

#### Assessment of publication bias

The publication bias using visual assessment of the funnel’s plots is presented in Supplementary Figures S1–S5.

## Discussion

Overall, we observed inconsistent epidemiological evidence that pyrethroid exposure led to a reduction in human semen quality. Pyrethroid exposure significantly reduced sperm motility across the studied populations and following subgroup analyses among men from Poland and the United States, however, it did not significantly alter other conventional semen parameters evaluated. However, in the region-stratified analyses, it was observed that pyrethroid significantly reduced sperm count and sperm concentration among men in Japan and Poland respectively. Surprisingly, an increase in sperm concentration was observed in men in the United States. Overall, these findings revealed that pyrethroid exposure has significant potential deleterious effects on sperm quality, especially sperm motility.

These findings are in agreement with the report of Young H. A. et al. (2013) that revealed an inconsistent effect of 3-phenoxy benzoic acid (3PBA), a pyrethroid metabolite, on human semen quality but an increased rate of aneuploidy with cis-3-(2,2-dichloro vinyl)-2,2-dimethylcyclopropane carboxylic acid (CDCCA) and trans-3-(2,2-dichlorovinyl)-2,2-di methylcyclopropane carboxylic acid (TDCCA) exposure. However, these do not align with the findings of Meeker J. D. et al. (2008) who demonstrated that pyrethroid exposure was associated with increased urinary pyrethroid metabolites and reduced semen quality in humans. Our present findings also do not align with the report of Radwan et al. (2014a) which showed that CDCCA and TDCCA reduced sperm concentration and circulating testosterone levels and increased abnormal sperm morphology.

In agreement with the present findings, Xia et al. (2008) revealed an association between 3PBA levels in the urine and sperm concentration, while ejaculate volume and sperm motility were weakly or non-significantly associated with 3PBA. Although the study of Bian et al. (2004) showed that fenvalerate induced sperm DNA damage, it also failed to show a significant alteration in sperm concentration and motility. Kamijina et al. (2004) revealed inconsistent effects as well, including reduced sperm motility following pyrethroid exposure in summer but not in winter, while sperm count and concentration were unaltered in either season (Kamijina et al., 2004). Similarly, Xia et al. (2004a) revealed the pyrethroid increased abnormal sperm morphology and induced sperm DNA damage, surprisingly ejaculate volume, sperm concentration, and sperm motility were not affected.

It is worth noting that most of the studies that reported a significant alteration in semen quality in pyrethroid-exposed men are non-controlled cross-sectional studies, whereas the studies that reported a non-significant change or no alteration at all are controlled studies. Possible explanations for the variations observed in the results from the controlled and non-controlled studies concerning the impact of pyrethroid exposure on semen quality might be due to the study design. Appropriate age-matched control could have formed a basis for proper comparison. Also, since the acute toxicity of synthetic pyrethroids to mammals is low, and non-occupational and low levels of exposure to environmental toxicants may exert a weak effect (Simbo et al., 2000; Olsson et al., 2002), the duration and level of exposure, and not just the urinary concentrations of metabolites are important. Hence, the level of exposure in the studied population likely influenced the outcome.

Nonetheless, there is a biological plausibility that pyrethroid exposure lowers semen quality and by extension induces male subfertility/infertility. Experimental studies in animal models have demonstrated convincing evidence linking pyrethroid exposure with reduced sperm quality; however, it is important to translate findings from animal models to humans with caution. Ravula and Yenugu (2021) revealed that exposure to a mixture of pyrethroids resulted in reduced sperm count and in the expression of genes that control gamete cell production. This was accompanied by impaired capacitation and acrosome reaction. In an *in vitro* study using sperm cells collected from Sprague Dawley rats, permethrin and cypermethrin reduced sperm motility in a dose-dependent and time-dependent fashion but 3-BPA did not, suggesting that perhaps, not all pyrethroids may exert the same effect on sperm parameters. The effect of cypermethrin has been linked with the induction of oxidative stress via ERK1/2-mediated mitochondrial dysfunction, increased reactive oxygen species generation, and ER stress (Wang et al., 2009).

Stewart et al. (2016) also demonstrated that exposure of sperm cells collected from a Bull to pyrethrin and cyfluthrin (24 mL cyfluthrin 1% solution (average dose 0.3 mg cyfluthrin/kg BW; 16 mL/1,000 sq. ft. of pyrethrin) for 18 weeks did not alter sperm motility. This further highlights the fact that different pyrethroids may exert dissimilar effects on sperm parameters. In another *in vitro* study using spermatozoa from mice, bifenthrin reduced sperm motility and kinematic variables as well as capacitation reaction (Bae and Kwon, 2021). The effect of bifenthrin was demonstrated to be via suppression of intracellular ATP levels and modulation of PKA activity, leading to the downregulation of protein tyrosine phosphorylation (Bae and Kwon, 2021). Deltamethrin has also been shown to reduce epididymal sperm motility, viability, and count, and increase the percentage of abnormal morphology in rodents (Abdallah et al., 2010; Ben Slima et al., 2013; Ben Slima et al., 2017). Administration of permethrin also impaired spermatogenesis in rats (Issam et al., 2011) possibly, leading to reduced sperm count.

**FIGURE 7:**
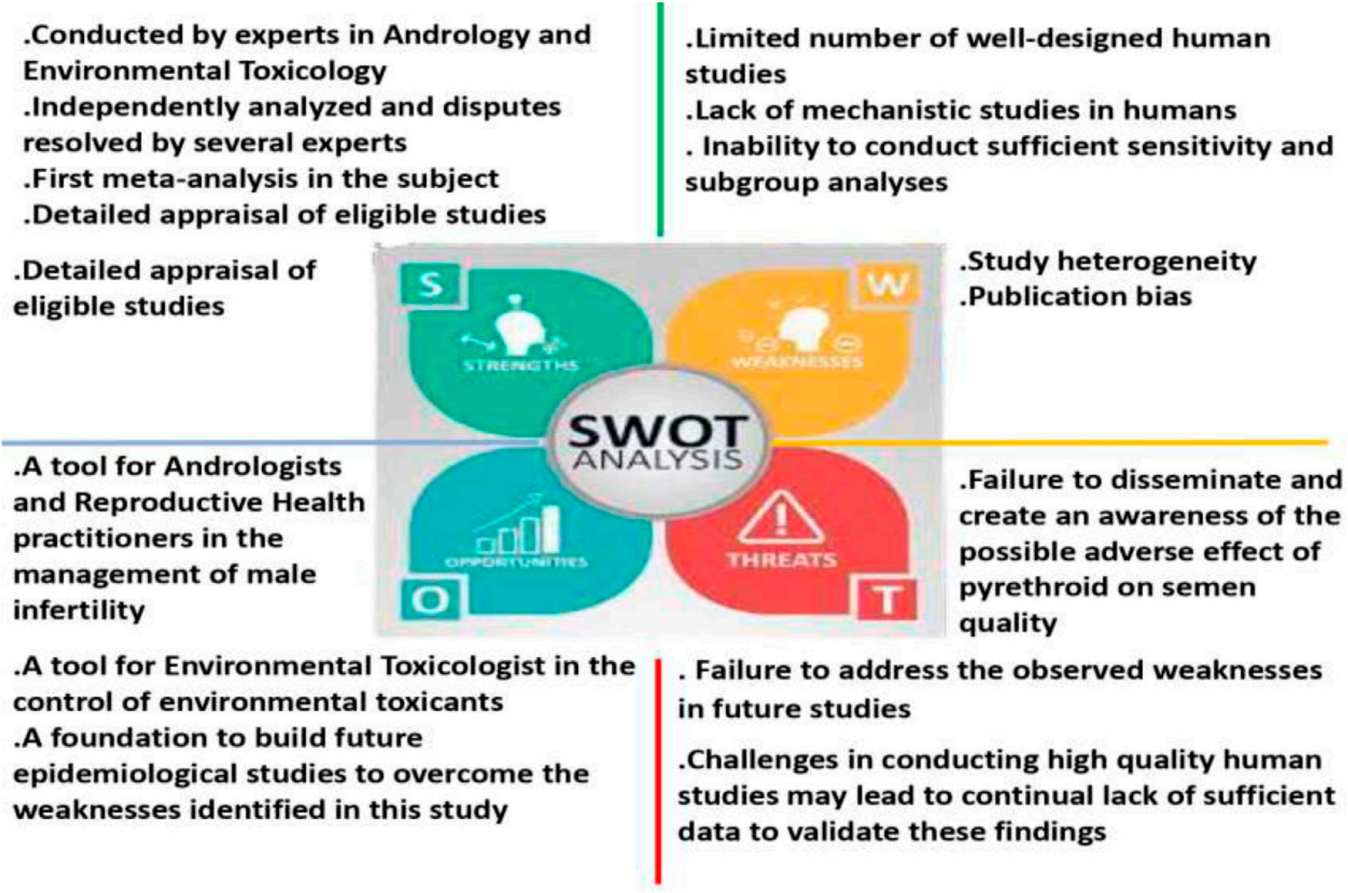
The strengths, weaknesses, opportunities, and threats (SWOT) analysis of the present meta-analysis.

Although the disparity observed in various studies and in comparison with the findings of the present meta-analysis may be attributed to exposure of the subjects to different pyrethroids which may exert different effects on sperm cells, duration and levels of exposure, and the route of exposure; pyrethroids have also been reported to be rapidly metabolized in mammals by the cleavage of the central ester linkage (Dorman and Beasley, 1991), yielding several endocrine disrupting metabolites that are more toxic than the parent compound (Tyler et al., 2000). Potentially, the rapid metabolism of pyrethroids leads to lower pyrethroid concentrations in the circulation and increased levels in the urine, thus curtailing the adverse effects of pyrethroids. Finally, we cannot completely rule out the possible detrimental effects of pyrethroids on semen quality; however, well-designed epidemiological studies with sufficient sample size are pertinent in exploring the impact and associated mechanisms of pyrethroids on semen quality. Despite the robust evaluation of the available data in the present study, there are some limitations. The Strengths, Weaknesses, Opportunities, and Threats of the current study are presented in Figure 7. The majority of the included studies were of moderate RoB and certainty of evidence. In addition, most of the included studies (eight) were studies that evaluated the effect of environmental pyrethroid exposure on semen quality and without controls. This is a reflection of the quality of the included studies and influenced the outcome of this meta-analysis. It is also important to note that despite the large pool of studies that were initially collected, only twelve were eligible for inclusion, indicating a scarcity of data on the impact of pyrethroid on human sperm quality. The low number of included studies might have negatively impacted the outcome of this study as it tends to reduce the pooled sample size and limited sensitivity and subgroup analyses.

Overall, this systematic review and meta-analysis found inconsistent epidemiological evidence that pyrethroid exposure lowered human semen quality. Although it was observed that sperm motility was significantly reduced and sperm count and concentration were altered following a region-stratified subgroup analysis, thus posing a threat to male fertility; these findings are not sufficient to conclude that pyrethroid reduces human semen quality. Future studies focusing on the assessment of semen quality in pyrethroid-exposed men in their reproductive age, particularly at varying known levels of exposure, and employing prospective cohort studies or controlled cross-sectional designs would be valuable to male reproductive health outcomes.

## Data Availability

The original contributions presented in the study are included in the article/Supplementary Material, further inquiries can be directed to the corresponding author.
