# Supplementary material for: Does pyrethroid exposure lower human semen quality? a systematic review and meta-analysis
Source: Front Toxicol. 2024 May 30;6:1395010. doi: 10.3389/ftox.2024.1395010 (PMC11196980; doi:10.3389/ftox.2024.1395010)
Supplement: Supplementary file 1 [file DataSheet1.PDF]

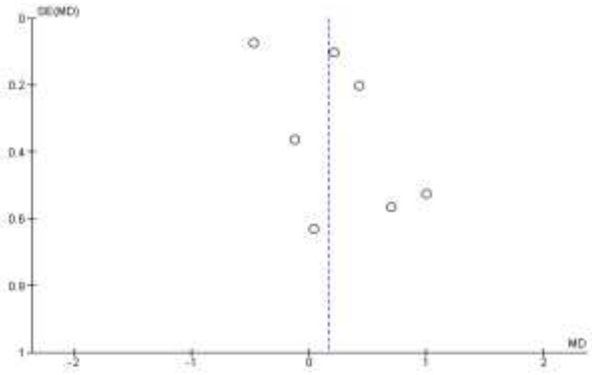

A.

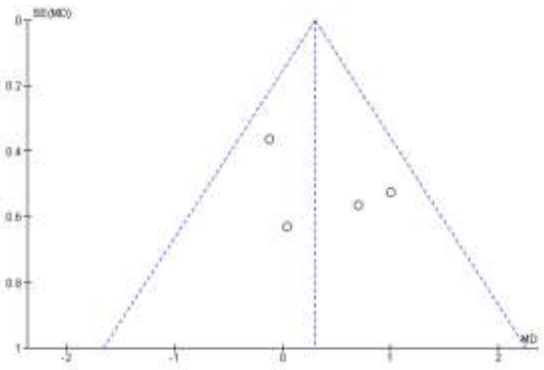

B.

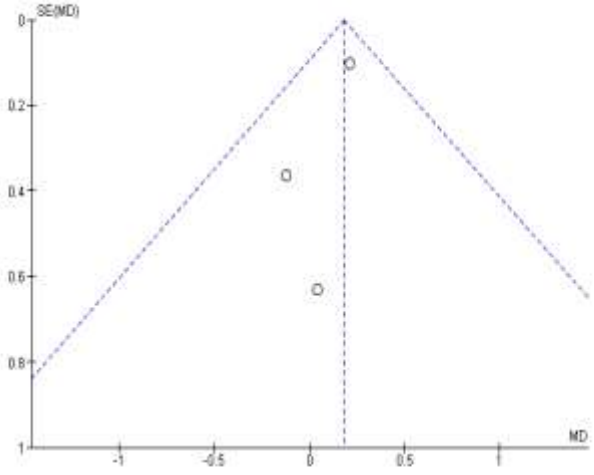

C.

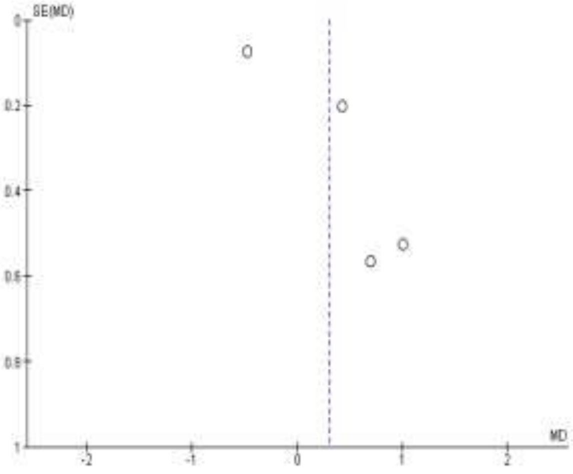

D.

Supplementary F1: Funnel's plot showing the publication bias of studies included for ejaculate volume after meta-analysis (A), sensitivity analysis (B), subgroup analysis among men in China (C), and subgroup analysis among men in Japan (D).

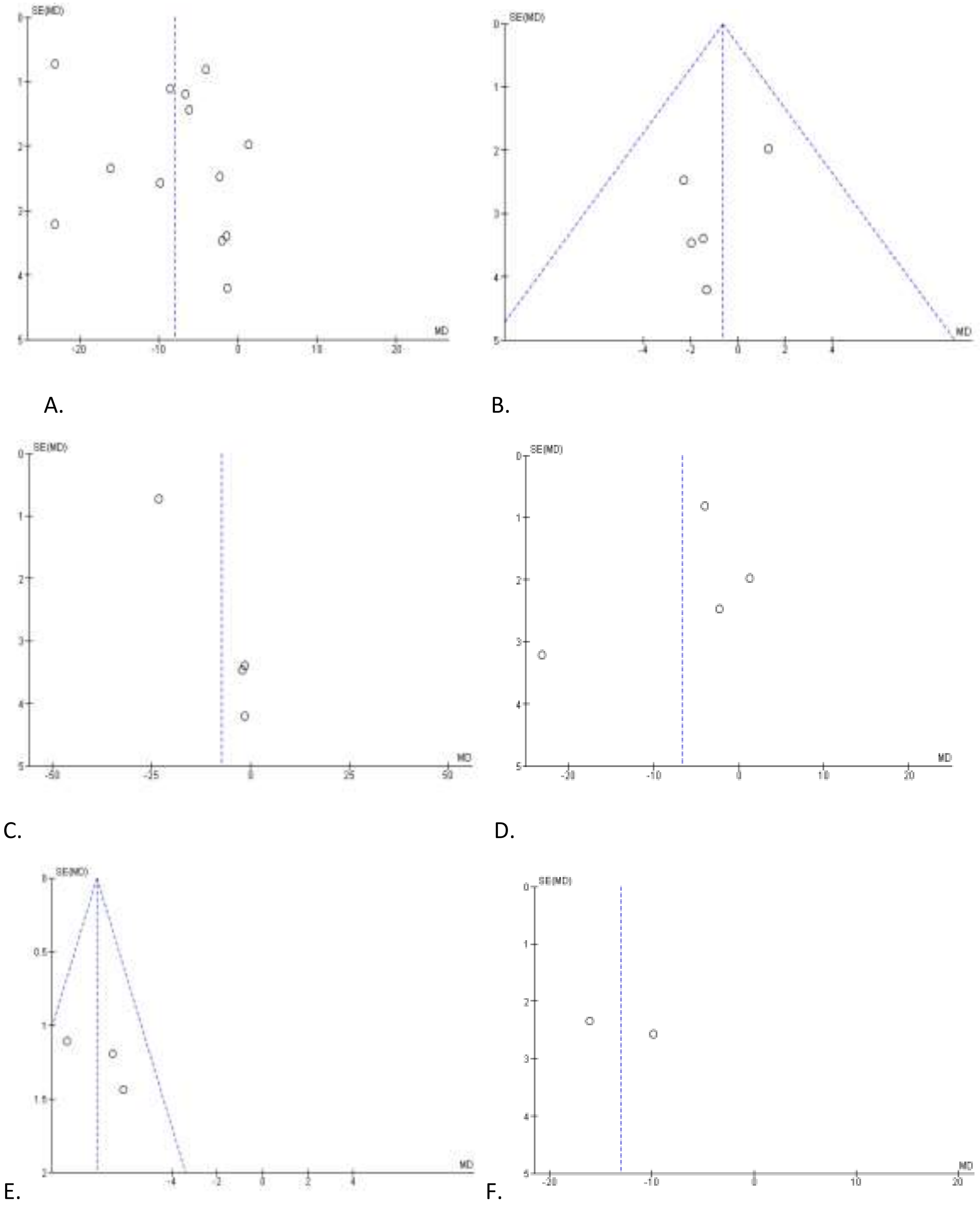

Supplementary F2: Funnel's plot showing the publication bias of studies included for sperm motility after meta-analysis (A), sensitivity analysis (B), subgroup analysis among men in China (C), subgroup analysis among men in Japan (D), , subgroup analysis among men in Poland (E), and subgroup analysis among men in USA (F).

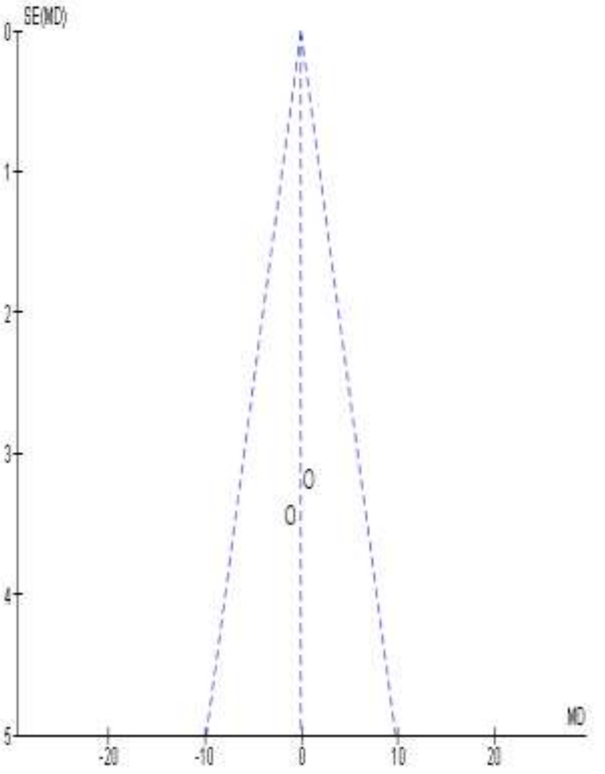

Supplementary F3: Funnel's plot showing the publication bias of studies included for sperm viability

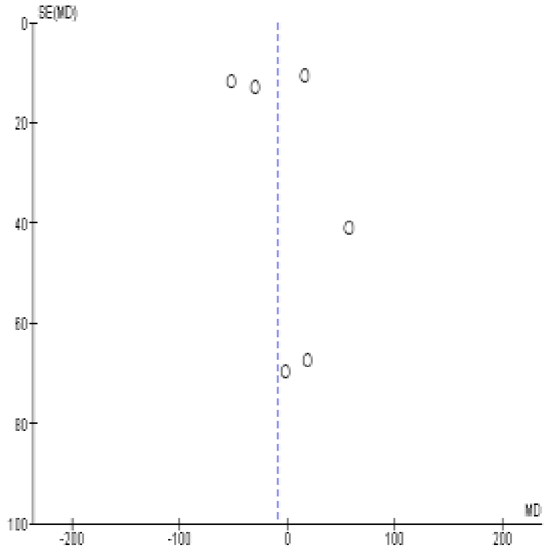

A.

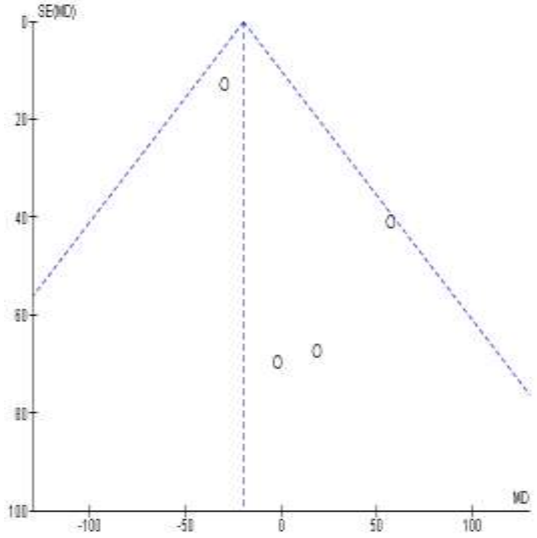

B.

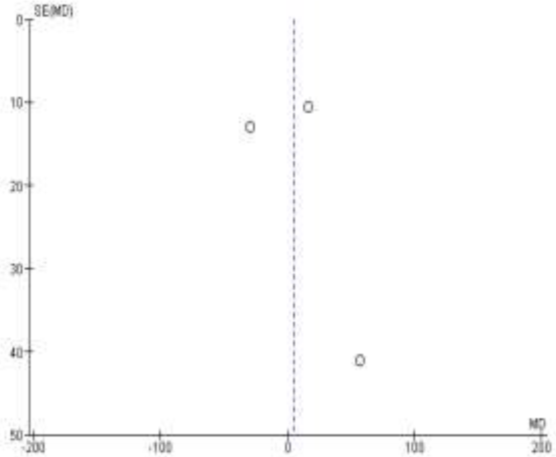

C.

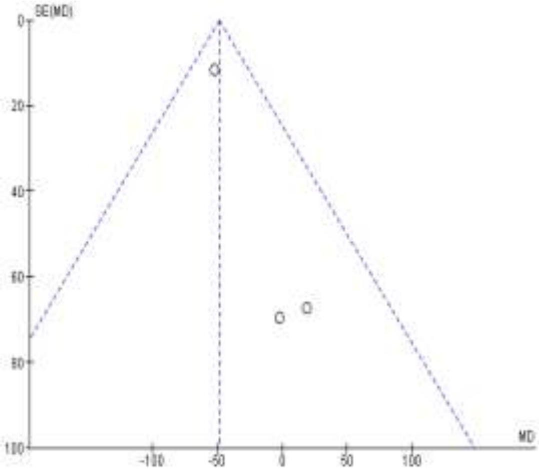

D.

Supplementary F4: Funnel's plot showing the publication bias of studies included for sperm count after meta-analysis (A), sensitivity analysis (B), subgroup analysis among men in China (C), and subgroup analysis among men in Japan (D).

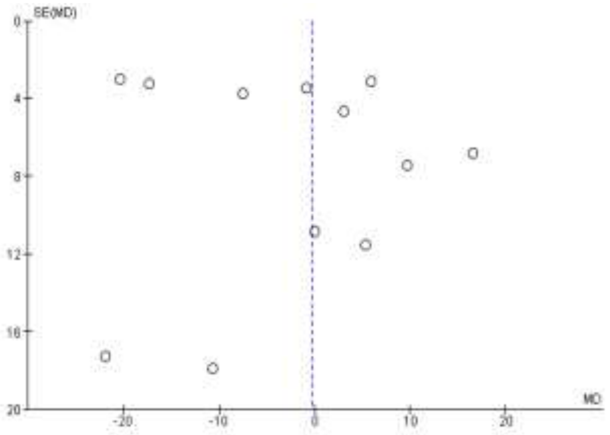

A.

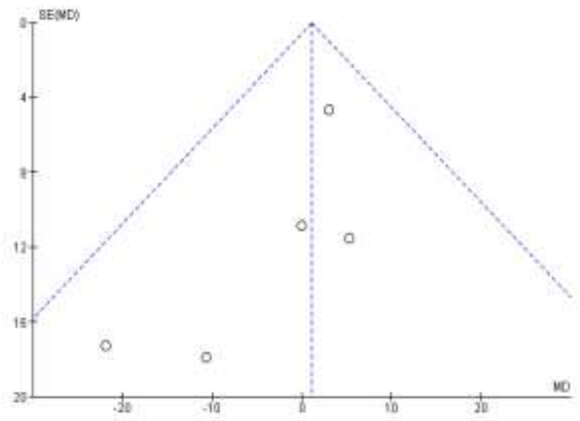

B.

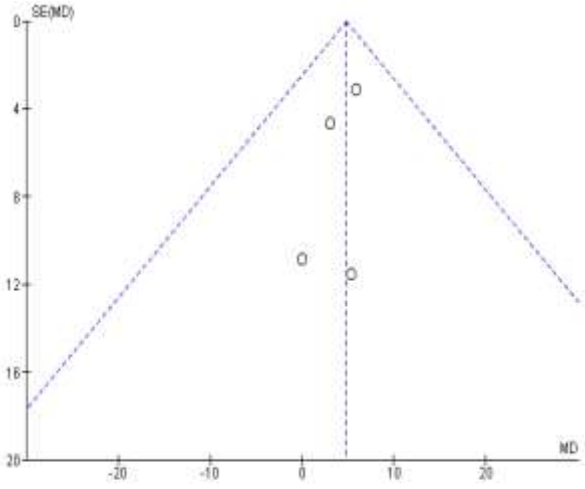

C.

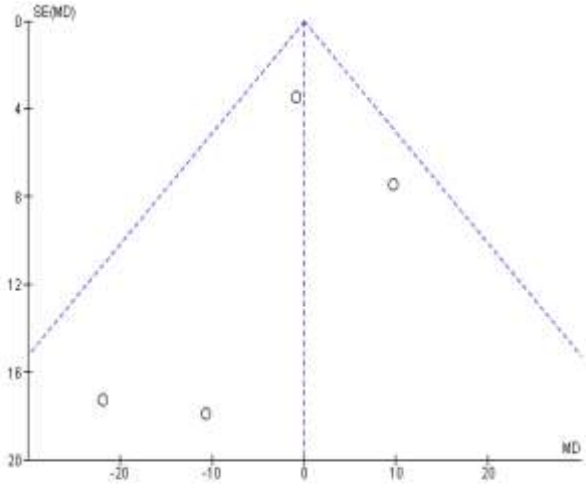

D.

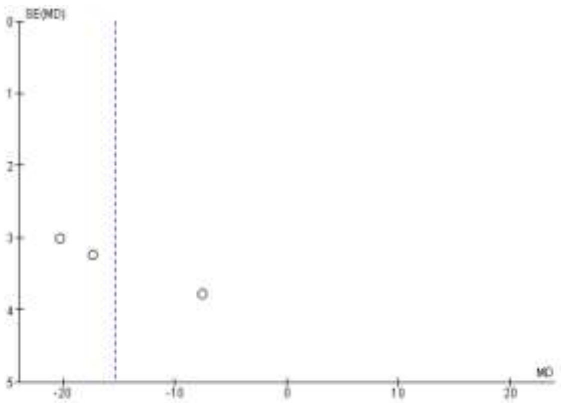

E.

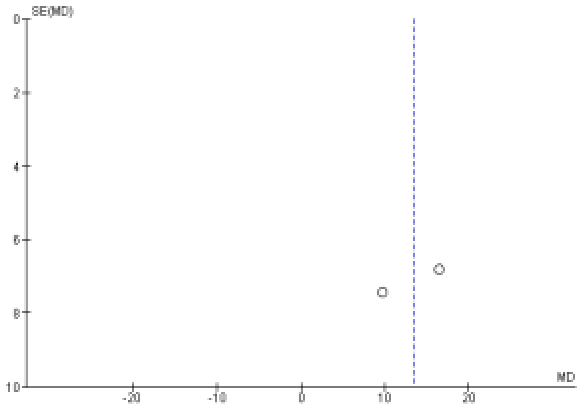

F.

Supplementary F5: Funnel's plot showing the publication bias of studies included for sperm concentration after meta-analysis (A), sensitivity analysis (B), subgroup analysis among men in China (C), subgroup analysis among men in Japan (D), , subgroup analysis among men in Poland (E), and subgroup analysis among men in USA (F).
